# Malignant Glomus Tumour: A Case Report and Review of the Literature

**DOI:** 10.1080/1357714031000081207

**Published:** 2003-06

**Authors:** Annarosaria De Chiara, Gaetano Apice, Stefano Mori, Giustino Silvestro, Simona N. Losito, Gerardo Botti, Vito Ninfo

**Affiliations:** 1 Department of Pathology Istituto dei Tumori di Napoli ‘G. Pascale’ di Napoli Via M. Semmola Napoli 80131 Italy; 2 Division of Medical Oncology B Istituto dei Tumori di Napoli ‘G. Pascale’ di Napoli Napoli Italy; 3 Division of Surgical Oncology B Istituto dei Tumori di Napoli ‘G. Pascale’ di Napoli Napoli Italy; 4 Division of Radiotherapy Istituto dei Tumori di Napoli ‘G. Pascale’ di Napoli Napoli Italy; 5 Department of Pathology Facoltà di Medicina e Chirurgia di Padova Padova Italy

## Abstract

*Purpose:* Glomus tumours are characteristically benign solitary tumours. At our knowledge, about 23 reports are present in literature regarding the malignant counterpart, but only a minority developed metastases. We describe a locally aggressive glomus tumour with lymphnode metastasis.

*Patient:* The patient was a 40 year-old man presenting a 1.5-cm lesion on the right wrist incompletely excised and a recurrent tumour, 4 × 2 cm in size, removed after 9 months, for which he received radiotherapy. After 2 years he developed an axillary lymphnode metastasis.

*Results:* Histologically, both tumours (primary and metastasis) were similar. There were sheets and nests of uniform small cells with scant eosinophilic cytoplasm and round to polygonal nuclei; there was some degree of pleomorphism and the mitotic index was high (up to 18 m/10 HPF). The tumour cells were positive for vimentin and smooth muscle actin, but negative for desmin, NSE, Factor VIII, chromogranin, cytokeratin. Remarkably, in the primary, the cells strongly expressed p53 (70%) and MIB-1 (35%).

*Discussions:* In many reported malignant cases, the histology of the tumour cells suggested that they were malignant, yet the clinical course has been benign. Carefully reviewing the literature, it seems that actually we have enough histological criteria to identify the cases with biological adverse outcome. Those unfortunate cases behave as high grade sarcomas and therefore may deserve an aggressive therapeutic treatment.


Sarcoma, June 2003, VOL. 7, NO. 2, 87–91

Taylor & Francis healthsciences

CASE REPORT

### Malignant glomus tumour: a case report and review of the literature*

ANNAROSARIA DE CHIARA[1], GAETANO APICE[2], STEFANO MORI[3], GIUSTINO SILVESTRO[4], SIMONA N. LOSITO[1], GERARDO BOTTI[1] & VITO NINFO[5]

[1]*Department of Pathology,* [2]*Division of Medical Oncology B,* [3]*Division of Surgical Oncology B and* [4]*Division of Radiotherapy, Istituto dei Tumori di Napoli 'G. Pascale' di Napoli, Napoli, Italy &* [5]*Department of Pathology Facoltà di Medicina e Chirurgia di Padova, Padova, Italy*

**Abstract**

*Purpose:* Glomus tumours are characteristically benign solitary tumours. At our knowledge, about 23 reports are present in literature regarding the malignant counterpart, but only a minority developed metastases. We describe a locally aggressive glomus tumour with lymphnode metastasis.

*Patient:* The patient was a 40 year-old man presenting a 1.5-cm lesion on the right wrist incompletely excised and a recurrent tumour, $4 \times 2$ cm in size, removed after 9 months, for which he received radiotherapy. After 2 years he developed an axillary lymphnode metastasis.

*Results:* Histologically, both tumours (primary and metastasis) were similar. There were sheets and nests of uniform small cells with scant eosinophilic cytoplasm and round to polygonal nuclei; there was some degree of pleomorphism and the mitotic index was high (up to 18 m/10 HPF). The tumour cells were positive for vimentin and smooth muscle actin, but negative for desmin, NSE, Factor VIII, chromogranin, cytokeratin. Remarkably, in the primary, the cells strongly expressed p53 (70%) and MIB-1 (35%).

*Discussions:* In many reported malignant cases, the histology of the tumour cells suggested that they were malignant, yet the clinical course has been benign. Carefully reviewing the literature, it seems that actually we have enough histological criteria to identify the cases with biological adverse outcome. Those unfortunate cases behave as high grade sarcomas and therefore may deserve an aggressive therapeutic treatment.

**Key words:** *glomus tumour, malignant, arm*

### Introduction

Glomus tumours (GT) are relatively uncommon lesions with an estimated incidence of about 1% among soft tissue tumours.[1] Usually they occur as solitary or even multicentric lesions, in the deep dermis or subcutis of the upper or lower extremity, with the most common location in the subungual region of the finger.[1] Unusual locations are also reported, including mediastinum and respiratory tract,[2–4] gastrointestinal tract,[5] gynecological regions.[1]

Glomus tumours are benign in the overtly majority of the cases. However, glomus tumours can show aggressive and/or malignant clinical and histological features. These atypical glomus tumours were classified in three different categories based on architectural and histological features:[6] locally infiltrative glomus tumours (LIGT), glomangiosarcoma arising within a glomus tumour (GABG), and *de novo* glomangiosarcoma (GADN). Following the morphological criteria, we were able to find 23 reports of malignant cases in literature.[1–4, 6–24] Since most of them did not metastasize, it is difficult to accept all of them as malignant tumours in the fullest meaning of the word and some cases of local recurrence probably represent persistence of the tumour following inadequate excision.[1] A malignant counterpart has been strongly questioned, since it was stated that "the malignant transformation is such a rare event that it can be dismissed from a practical point of view".[1] However, reviewing the literature carefully, some metastatic cases were evident, often widespread in a short time, proving that the malignant glomus tumours (MGT) do really exist. A similar conclusion has been drawn from the authors of a recently published paper studying their large consultation files.[24]

*This manuscript was presented at the 2nd World Congress of the World Federation of Surgical Oncology Society–WFSOS, Napoli, Italy, 19–22 September 2001.

Correspondence to: Anna De Chiara, Servizio di Anatomia Patologica, Istituto dei Tumori 'G. Pascale', Via M. Semmola, 80131 Napoli, Italy. Tel: +39-81-5903359; Fax: +39-81-5461688; E-mail: annadechiara@hotmail.com

ISSN 1357-714X print/1369-1643 © 2003 Taylor & Francis Ltd
DOI: 10.1080/1357714031000081207

We describe a *de novo* glomangiosarcoma (GADN) metastasizing to an axillary lymphnode after 2 years. Our findings are similar to the morphological and the prognostic criteria proposed by Folpe *et al.* in their extensive work.[24]

### Clinical history

The patient was a 40-year-old man presenting a 1.5-cm lesion on the right wrist incompletely excised and interpreted as hemangiopericytoma at another institution. After 9 months he was referred to Napoli NCI for a recurrent tumour. A $5 \times 4$-cm biopsy showed a gray, non-encapsulated nodule, $4 \times 2$ cm in size, located in the deep dermis. At this time, we also reviewed the slides from the original tumour. An exhaustive clinical work up revealed no metastases. Because of the histological diagnosis of a low-grade sarcoma, the patient received a total dose of 56 Gy. After 2 years, a FNAC on an enlarged axillary lymphnode yielded a positive result. A complete axillary dissection was performed which revealed diffuse metastasis to one out of 29 lymphnodes. The patient then received chemotherapy (epirubicin 120 mg/m$^2$, ifosfamide 9 g/m$^2$).

Histologically, the primary and the recurrent tumour were both remarkably similar. There were sheets and nests of uniform round or polygonal cells infiltrating the reticular layer of the dermis with distinct cell borders and scant eosinophilic cytoplasm; there was some degree of pleomorphism and the mitotic index was high (up to 5 mitoses/10 HPF) (Fig. 1). A spindle cells growth was focally present (Fig. 2) and an hemangiopericytoma pattern was also focally evident (Fig. 3). Moreover, the glomocytes formed subendothelial masses that protruded into the lumens of the venous channels and small tumour nodules were clearly intravascular (Fig. 4). No structure resembling a glomus body was observed. Cytoplasmic positivity for vimentin and for smooth muscle actin was present (Fig. 5). A pericellular staining around cells with collagen type IV was also seen. Other markers such as desmin, NSE, chromogranin, cytokeratin, EMA, Factor VIII were all negative. Remarkably, the cells strongly expressed p53 (70%) and MIB-1 (35%). Based on these features, the case was considered to be a malignant glomus tumour, that is, a glomangiosarcoma arising *de novo*. The lymphnode showed similar histological findings with some very large cavernous vessels always surrounded by glomus cells and cystic degeneration filled with proteinaceous material (Fig. 6).

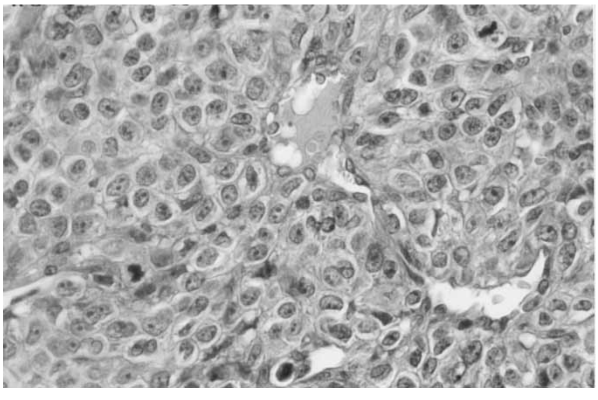

**Fig. 1.** *Neoplastic cells show round to oval nuclei with very distinct cell borders. Note the small but evident nucleoli and the mitotic figure (H&E, × 20).*

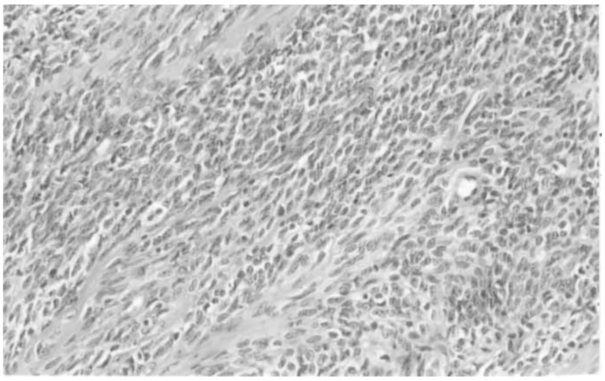

**Fig. 2.** *Focally an hypercellular spindle cells pattern is well evident (H&E, × 10).*

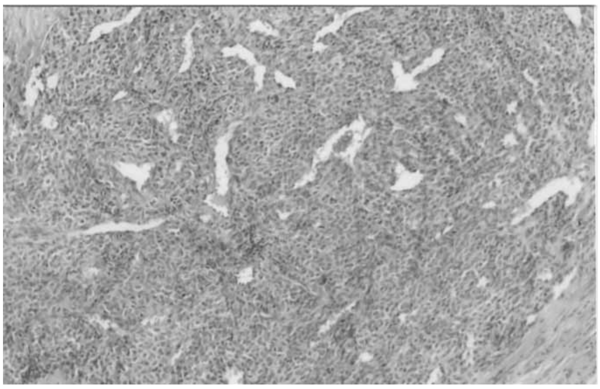

**Fig. 3.** *A prominent vascular component is always present with features sometimes reminiscent of hemangiopericytoma (H&E, × 4).*

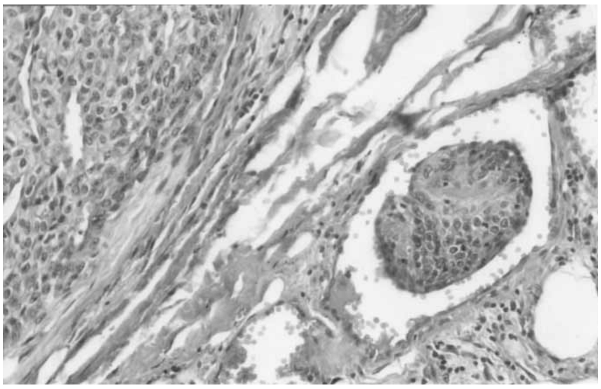

**Fig. 4.** *The tumour (on left) presents clearly vascular space involvement (on right) (H&E, × 10).*

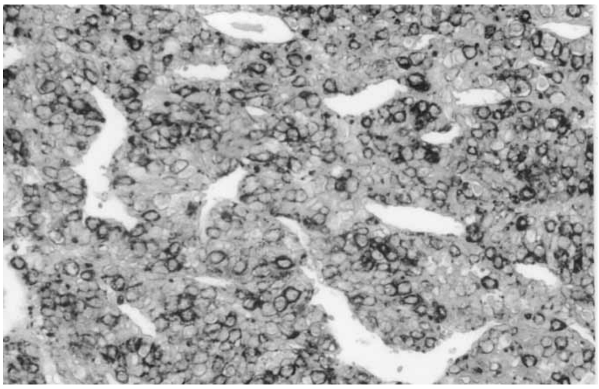

**Fig. 5.** *The neoplastic cells present strong smooth muscle actin expression (immunoperoxidase, × 10).*

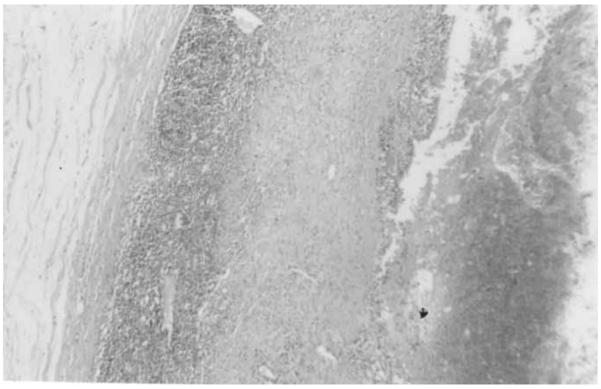

**Fig. 6.** *The lymph node sections show a peripheral thin rim of uninvolved tissue (on the left) and large hemorrhagic space surrounded by neoplatsic cells similar to primary (H&E, × 4).*

### Discussion

The glomus tumour is a distinct neoplasm composed of perivascular cells resembling the modified smooth muscle cells of the normal glomus body.[1] The glomus body is a specialized form of arterio-venous anastomosis that serves in thermal regulation. It is located in the stratum reticularis of the dermis and is most frequently encountered in the subungual region, the lateral areas of the digits, and the palm. Is therefore in those areas that the glomus tumour is mainly found. But the tumour can also be encountered in other sites where the normal glomus body may be sparse or even absent,[1] such as mediastinum and respiratory tract,[2–4] gastrointestinal tract,[5] gynecological regions.[1] Usually they are solitary, painful, well-circumscribed tumours, cured by simple excision. Multiple forms do also occur. They tend to be asymptomatic, rarely subungual, and acquired during the childhood.

Histologically, the glomus tumour is a well-circumscribed lesion consisting of convoluted capillary-sized vessels surrounded by glomus cells in a hyalinized or myxoid stroma. The tumour may have a highly vascular pattern reminiscent of a hemangiopericytoma. The cells are monomorphous with a rounded, regular shape and somewhat cohesive, giving them an epithelioid appearance.

Some cytologically benign tumours are locally infiltrative and tend to recur after simple excision.[6] They are usually larger and more deeply located than the conventional ones. They may show a more extensive solid growth pattern or cavernous glomangioma pattern. These locally infiltrative glomus tumours (LIGT) may therefore need a more complete excision. The recognition of a malignant counterpart has been controversial. There are two described categories: the first is a cytologically malignant tumour arising and merging with a typical glomus tumour, designated glomangiosarcoma in a benign glomus (GABG)[14]. In these cases the malignant component shows mitotic figures and cytologic atypia[6] or even has features of a spindle cell sarcoma.[1,15,17] Such cytological atypia has also been interpreted simply as an epitheliod change in an otherwise typical glomus tumour similar to nuclear variability present in other biologically benign tumour such as collagenous fibroma of the skin, ancient schwannoma and symplastic leiomyoma.[25] Since none of these cases metastasized, the existence of a MGT has strongly been questioned.[1] However, the malignant areas prove to show molecular abnormalities such as increased bcl-2 and p53 expression, not present in the benign component or in benign glomus tumours (GT) as internal control.[17,26] These results suggest that MGT may have a potentially more aggressive behavior than benign GTs.

The second malignant group, and more difficult to recognize, is *de novo* glomangiosarcoma (GADN), in which a benign glomus component cannot be identified. It is composed by undifferentiated small round or oval cells, with vesicular nuclei, often prominent nucleoli and scant cytoplasm, which grow in large sheets; the mitotic index varies but it is always well appreciated.[2,3,6,8,9,11,15,18,19,22] Thus the tumour must be distinguished from other round cell sarcomas, or metastatic carcinoma because of the overall epithelioid appearance. However, the diagnostic clue is that the cell population and the architecture closely resemble those of the benign glomus. As a matter of fact, in at least some areas, the tumour cells invest and intimately relate to vessels like its benign counterpart. The immunostaining pattern for glomus tumours (actin positive, epithelial markers negative) are helpful in the differential diagnosis. In addition, glomus tumours show prominent intercellular reticulin, laminin and collagen type IV staining.[23,27]

Confluent zones of necrosis are only occasionally present.[23,24] A typical feature is indeed the presence of pseudocysts of varying size often filled of proteinaceous material, which seem to be the result of cystic degeneration.[3,6,18,23] Most of the single cases diagnosed as potentially malignant glomus tumours fall in the GADN category. However, only eight of them metastasized.[7,8,10–12,18,22,23] In the only large published series[24] based on 35 potentially malignant cases, the overall metastatic rate for 21 patients with follow-up was 38% (eight out of 21 patients). The authors found that the adverse outcome was notably related to deep location, size larger than 2 cm, and the presence of atypical mitotic figures. However, some other features, including necrosis, mitotic activity of more than five mitoses/50 HPF, and combination of high nuclear grade and high mitotic activity showed a trend toward but did not achieve statistical significance.[24] As a matter of fact, our patient, as well as case no. 25 of their series and the patient reported by Watanabe *et al.*,[22] showed high nuclear grade and high mitotic activity and, even though superficial in location, metastasized.

Vascular space involvement was observed in 10 cases,[11,24] and lymphnode metastases were present in at least three patients.[11,18,24] Our case, interpreted as GADN, also showed some intravascular tumour nodules and presented a metastatic axillary lymphnode after 2 years, despite radiotherapy having achieved a local control of the disease. Therefore we could hypothesize that the intavascular invasion is the key feature for the metastatic potential of the MGT. However, intravascular spread was also described in one GABG of the stomach[5] and in one GADN of the right thigh[19] that did not show recurrence or metastasis after 7 and 57 months after surgery, respectively. An alternative explanation is that the intravascular spread in glomus tumour is analogous to that observed in intravascular leiomyomatosis.[5]

MGTs were usually believed low-grade sarcomas.[6,23] However, five out of eight metastatic GADN (two of which located in the lung) showed widespread metastases[10,11,18,22,23] in a few months and rapidly died of the disease[10,18,23] despite chemotherapeutic regimens. In the series reported by Folpe *et al.*,[24] of the eight patients who developed metastatic disease, six were dead in less than 3 years.

In conclusion, the malignant glomus tumours do really exist and we have enough histological criteria to identify them. They are probably high grade sarcomas and should be treated as such.

### Acknowledgements

The authors wish to thank Mr. L. Iodice and Mr. A. Barbato for their technical assistance.

### References

1. Enzinger FM, Weiss SW. *Soft Tissue Tumors*. St. Louis, MO: CV Mosby, 1995: 701–13.
2. Choi TJ, Yang KH, Gang SJ, Kim BK, Kim SM. Malignant glomus tumor originating in the superior mediastinum: an immunohistochemical and ultrastructural study. *J Korean Med Sci* 1991; 6: 157–63.
3. Hirose T, Hasegawa T, Seki K, Yang P, Sano T, Morizumi H, Tsuyuguchi M. Atypical glomus tumor in the mediastinum: a case report with immunohistochemical and ultrastructural studies. *Ultrastruct Pathol* 1996; 20: 451–6.
4. Gaertner E, Steinberg DM, Huber M, Hayashi T, Tsuda N, Askin FB, Bell SW, Nguyen B, Colby TV, Nishimura SL, Miettinen M, Travis WD. Pulmonary and mediastinal glomus tumors. Report of five cases including a pulmonary glomangiosarcoma: a clinicopathologic study with literature review. *Am J Surg Pathol* 2000; 24: 1105–14.
5. Haque S, Modlin IM, West AB. Multiple glomus tumors of the stomach with intravascular spread. *Am J Surg Pathol* 1992; 16: 291–9.
6. Gould EW, Manivel JC, Albores-Saavedra J, Monforte H. Locally infiltrative glomus tumors and glomangiosarcomas. *Cancer* 1990; 65: 310–8.
7. Kirshbaum GB, Teitelman SL. Malignant tumor of the greater omentum simulating a glomangiosarcoma. *Arch Pathol* 1939; 27: 95.
8. Lumley JS, Stansfeld AG. Infiltrating glomus tumor of lower limb. *Br Med J* 1972; 1: 484–5.
9. Anagnostou GD, Papademetriou DG, Toumazani MN. Subcutaneous glomus tumor. *Surg Gynecol Obstet* 1973; 136: 945–50.
10. Symmers WSC. Glomus tumor (Letter to the editor). *Br Med J* 1973; April 7: 50.
11. Mackay B, Legha SS, Pickler GM. Coin lesion of the lung in a 19-year-old male. *Ultrastruct Pathol* 1981; 2: 289–94.
12. Kreutz RW, Christensen REJ. Glomangiosarcoma with metastases to the maxilla. *Int J Oral Maxillofac Surg* 1987; 16: 116–8.
13. Perez-Alfonso R, Hevia Y, Planas-Giron G. Atypical glomus tumor. Report of a case. *Med Cutan Ibero Lat Am* 1987; 15: 256–8.
14. Aiba M, Hirayama A, Kuramochi S. Glomangiosarcoma in a glomus tumor: an immunohistochemical and ultrastructural study. *Cancer* 1988; 61: 1467–71.
15. Noer H, Krogdahl A. Glomangiosarcoma of the lower extremity. *Histopathology* 1991; 18: 365–6.
16. Daskalopoulou D, Maounis N, Kokalis G, Liodandonaki P, Belezini E, Markidou S. The role of fine needle aspiration cytology in the diagnosis of primary skin tumors. *Arch Anat Cytol Pathol* 1993; 41: 75–81.
17. Watanabe K, Hoshi N, Tsu-Ura Y, Suzuki T. A case of glomangiosarcoma. *Fukushima J Med Sci* 1995; 41: 71–7.
18. Brathwaite CD, Poppiti RJ. Malignant glomus tumor. A case report of widespread metastases in a patient with multiple glomus body hamartomas. *Am J Surg Pathol* 1996; 20: 233–8.
19. Hiruta N, Kameda N, Tokudome T, Tsuchiya K, Nonaka H, Hatori T, Akima M, Miura M. Malignant glomus tumor: a case report and review of the literature. *Am J Surg Pathol* 1997; 21: 1096–103.
20. Lopez-Rios F, Rodriguez-Peralto JL, Castano E, Ballestin C. Glomangiosarcoma of the lower limb: a case report with a literature review. *J Cutan Pathol* 1997; 24: 571–4.
21. Wetherington RW, Lyle WG, Sangueza OP. Malignant glomus tumor of the tumb: a case report. *J Hand Surg Am* 1997; 22: 1098–102.
22. Watanabe K, Sugino T, Kusakabbe T, Suzuki T. Glomangiosarcoma of the hip: a report of a highly aggressive tumor with widespread metastases. *Br J Dermatol* 1998; 139: 1097–101.
23. Gaertner E, Steinberg DM, Huber M, Hayashi T, Tsuda N, Askin FB, Bell SW, Nguyen B, Colby TV, Nishimura SL, Miettinen M, Travis WD. Pulmonary and mediastinal glomus tumors. Report of five cases including a pulmonary glomangiosarcoma: a clinicopathologic study with literature review. *Am J Surg Pathol* 2000; 24: 1105–14.
24. Folpe AL, Fanburg-Smith JC, Miettinen M, Weiss SW. Atypical and malignant glomus tumors: analysis of 52 cases, with a proposal for the reclassification of glomus tumors. *Am J Surg Pathol* 2001; 25: 1–12.
25. Pulitzer DR, Martin PC, Reed RJ. Epithelioid Glomus tumor. *Hum Pathol* 1995; 26: 1022–7.
26. Hegyi L, Cormack GC, Grant JW. Histochemical investigation into the molecular mechanisms of malignant transformation in a benign glomus tumor. *J Clin Pathol* 1998; 51: 872–4.
27. Ogawa K, Oguchi M, Yamabe H, Nakashima Y, Mamashima Y. Distribution of collagen IV in soft tissue tumors. An immunohistochemical study. *Cancer* 1986; 58: 269–77.
